# Blending In Situ Polyurethane-Urea with Different Kinds of Rubber: Performance and Compatibility Aspects

**DOI:** 10.3390/ma11112175

**Published:** 2018-11-02

**Authors:** Muhammad Tahir, Gert Heinrich, Nasir Mahmood, Regine Boldt, Sven Wießner, Klaus Werner Stöckelhuber

**Affiliations:** 1 Leibniz-Institut für Polymerforschung Dresden e.V., Hohe Straße 6, 01069 Dresden, Germany; tahir@ipfdd.de (M.T.); gheinrich@ipfdd.de (G.H.); boldt@ipfdd.de (R.B.); wiessner@ipfdd.de (S.W.); 2 Institut für Werkstoffwissenschaft, Technische Universität Dresden, Helmholtzstraße 7, 01062 Dresden, Germany; 3 Institut für Textilmaschinen und Textile Hochleistungswerkstofftechnik, Technische Universität Dresden, Hohe Straße 6, 01069 Dresden, Germany; 4 Faculty of Natural Sciences II (Chemistry, Physics and Mathematics), Martin Luther Universität Halle Wittenberg, 06120 Halle, Germany; nasir.mahmood@chemie.uni-halle.de

**Keywords:** elastomers, rubber blends, polyurethane-urea, in situ synthesis, compatibilization, structural/mechanical characterization

## Abstract

Specific physical and reactive compatibilization strategies are applied to enhance the interfacial adhesion and mechanical properties of heterogeneous polymer blends. Another pertinent challenge is the need of energy-intensive blending methods to blend high-tech polymers such as the blending of a pre-made hard polyurethane (-urea) with rubbers. We developed and investigated a reactive blending method to prepare the outstanding blends based on polyurethane-urea and rubbers at a low blending temperature and without any interfacial compatibilizing agent. In this study, the polyurethane-urea (PUU) was synthesized via the methylene diphenyl diisocyanate end-capped prepolymer and m-phenylene diamine based precursor route during blending at 100 °C with polar (carboxylated nitrile rubber (XNBR) and chloroprene rubber (CR)) and non-polar (natural rubber (NR), styrene butadiene rubber (sSBR), and ethylene propylene butadiene rubber (EPDM)) rubbers. We found that the in situ PUU reinforces the tensile response at low strain region and the dynamic-mechanical response up to 150 °C in the case of all used rubbers. Scanning electron microscopy reveals a stronger rubber/PUU interface, which promotes an effective stress transfer between the blend phases. Furthermore, energy filtered transmission electron microscopy (EFTEM) based elemental carbon map identifies an interphase region along the interface between the nitrile rubber and in situ PUU phases of this exemplary blend type.

## 1. Introduction

The blending of polymers is a useful route to develop new materials of practical importance. Various blending techniques including solution, melt, fine powder, latex, reactive and interpenetrating polymer network (IPN) processes are opted to produce homogeneous or heterogeneous polymer blends [1,2,3,4,5]. Commercially, melt blending procedures predominate due to the straightforward mechanical mixing of two or more polymers; however, in most cases, this requires a high mixing temperature and produces heterogeneous incompatible blends. Such blends require compatibilizing agents to promote interfacial adhesion, morphological stability and overall performance [6,7]. The melt blending procedures to prepare high performance heterogeneous blend systems is costly; therefore, a new blending method to mix hard polymers such as polyurethane-urea with soft polymers such as rubber was investigated.

Numerous studies have reported blending of various types of polyurethanes with rubbers. These blends are prepared by energy-intensive mixing processes and offer a very narrow temperature range of usage. Polyurethane anionomers and cationomers are melt-blended at around 170 °C with nitrile butadiene rubber and natural rubber. The prepared blends demonstrate an undesirable temperature-dependent decrease in dynamic mechanical response [8]. Ionomers appear to reinforce the rubber matrix at low temperatures; however, the reinforcing capability of ionomers fades away at around 100 °C. In another study, experimental evidence of compatibility in dynamically vulcanized blends of thermoplastic polyurethane and nitrile rubber is reported. The blends are prepared by a melt-blending method at a high temperature of 160 °C [9]. Another study discloses the method of blending a polyurethane adhesive with ethylene propylene diene (EPDM) rubber. The vulcanization of EPDM rubber and formation of polyurethane polymer of blend is realized during compression molding. The polyurethane phase retains its reinforcing capability only until 50 °C [10]. The wear resistance of hydrogenated nitrile rubber (HNBR) is shown to improve on blending with polyurethane adhesive composed of blocked polyisocyanate and polyol components. The hot melt PU adhesive is cured simultaneously with HNBR during vulcanization step. Here again, the melting of polyurethane phase limits the application temperature to 50 °C [11]. Silica loaded polyurethane precursor system is blended with nitrile rubber in another study [12]. The isocyanate end-capped prepolymer is reported to react with hydroxyl groups of silica surface during the vulcanization of nitrile rubber (NBR). It is revealed by dynamic tests that the dissipation of mechanical energy increases with the increasing fraction of polyurethane in blends. The layered silicates filled natural rubber and polyurethane based blends are produced by a distinct method of latex mixing. The microscopic and spectroscopic characterization of blend composites reveal that the polyurethane chains intercalated in layered silicates of the reported incompatible blend nanocomposites [13,14]. In another study, polyurethane-urea polymer and carboxylated nitrile butadiene rubber are solution blended. The dynamic-mechanical characterization of blends reveal softening of polyurethane-urea phase at around 50 °C for an excessive heat build-up in blends, which deteriorates the dynamic-mechanical response of the reported blends [15,16].

A linear polyurethane-urea is produced by the addition reactions of diisocyanates with macrodiols to create urethane moieties and additionally with short chain diamines to create urea moieties. Urea groups offer high density of hydrogen-bond to improve thermal stability and structural integrity of polyurethane-urea polymer. In a polyurethane-urea polymer, the macrodiols constitute soft segments (SS), which accumulate into soft domains and impart flexibility to the chains. The diisocyanate and diamine form hard segments (HS), which tend to phase separate from SS due to thermodynamic incompatibility into hard domains. The mechanical performance of a polyurethane-urea depends on its two-phase or phase-mixed structural morphology formed from soft and hard domains [17,18,19,20,21]. One-shot and pre-polymer techniques are used to synthesize a polyurethane-urea polymer. Relatively regular sequence of soft and hard segments is formed along the polymer chains when polyurethane-urea is synthesized via the prepolymer method [22]. 

In the present study, we investigated a reactive blending method wherein the polyurethane-urea was synthesized from its monomeric/pre-polymeric precursors during a simple mechanical mixing with both polar and nonpolar rubbers and attempted to benefit from the fact that the opted blending procedure is intrinsically compatibilizing and a low temperature mixing process [23,24]. Amongst the selected rubbers, carboxylated nitrile rubber (XNBR) and chloroprene rubber (CR) are of polar character due to the presence of acrylonitrile along with carboxylic and chloro moieties, respectively. Natural rubber (NR), styrene butadiene rubber (sSBR) and ethylene propylene butadiene rubber (EPDM) are non-polar elastomers due to the absence of any polar functional group.

## 2. Materials and Methods

### 2.1. Materials 

The solution styrene butadiene rubber (sSBR) of grade VSL 4526-0 HM was from Leverkusen, Lanxess, Germany. It is a copolymer consisting of styrene and butadiene monomers with a styrene content of 26 wt % and Mooney viscosity of 65 MU (ASTM D 1646). Natural rubber (NR) is the Standard Malaysian Rubber of grade SMR 10. The ethylene propylene diene (EPDM) rubber of grade EP G6850 is a terpolymer of ethylene, propylene and ethylidene norbornene monomer with a Mooney viscosity of 60 MU (ASTM D 1646) and was obtained from Leverkusen, Laxness, Germany. The chloroprene rubber (CR) is a sulfur-modified grade as Bayprene 611 with the Mooney viscosity of 35 ± 5 MU (ASTM D 1646) and of slight to medium crystallization tendency. CR was provided by Lanxess, Cologne, Germany. The carboxylated nitrile butadiene rubber (XNBR), a carboxylated version of nitrile rubber, is of grade X740 and from Lanxess, Cologne, Germany. It has an acrylonitrile content of 26.5 ± 1.5 wt % and the Mooney viscosity of 38 ± 4 MU (ASTM D 1646). 

4,4′-diphenylmethane diisocyanate (MDI) and poly (tetramethylene ether) glycol (PTMEG) based isocyanate terminated prepolymer precursor (MT2184) were acquired from Covestro Elastomers SAS (previously Baulé SAS), Romans-sur-Isère, France. MT 2184 has viscosity of 0.8 Pa·s at 80 °C. To prepare a premix, the prepolymer’s isocyanate contents of 8.55 wt % were used to set 1:1 stoichiometry with chain extender.
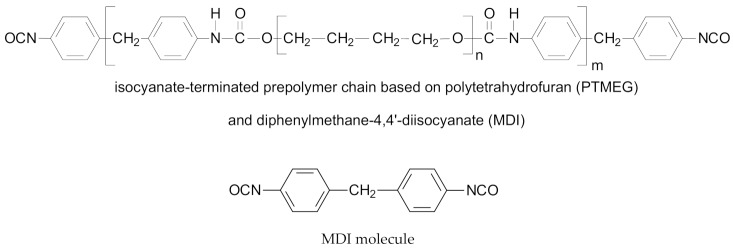


1,3-phenylenediamine (mPD) is about 99% pure, having a melting temperature of around 64 °C and was acquired from Sigma-Aldrich Co. LLC (St. Louis, MO, USA).

The stoichiometric quantities of mPD and MT2184 produce polyurethane-urea of 32.8 wt % hard segments.
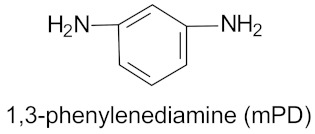


### 2.2. Methods 

#### 2.2.1. Cure Characteristics

The curing curves of compounds were obtained at 160 °C using a RPA Scarabaeus SIS V50 (Scarabaeus Mess-und Produktionstechnik GmbH, Langgöns, Germany).

#### 2.2.2. Mechanical Testing

The tensile testing of dumbbell shaped specimens was done according to the tensile testing standard DIN 53504. A preload of 0.2 N and a cross-head speed of 200 mm/min were the measurement parameters for testing on a tensile testing machine from Zwick 1456, Z010, Ulm, Germany.

#### 2.2.3. Scanning Electron Microscopy (SEM) Coupled with Energy Dispersive X-ray Analysis (EDX)

The specimens were cryo-fractured and sputter coated with carbon before scanning with ultra plus scanning electron microscope from Zeiss NTS (Oberkochem, Germany). The energy-dispersive X-ray analysis was performed with X-Flasch 5060F detector from Bruker nano GmbH (Berlin, Germany) to get elemental maps to distinguish the blend phases.

#### 2.2.4. Energy Filtered Transmission Electron Microscopy (EFTEM)

Thin sections of the blends were cut by ultramicrotome (Leica UC6/FC6, Leica Microsystems GmbH, Wetzlar, Germany) at −140 °C. The thin sections were stained with OsO_4_ and inspected with energy filtered transmission electron microscope (Libra200, Carl Zeiss Microscopy GmbH, Oberkochen, Germany) at an acceleration voltage of 200 kV.

#### 2.2.5. Dynamic Mechanical Analysis

Dynamic mechanical analysis (DMA) was done on specimen of 2 × 10 × 35 mm^3^ dimensions in the tensile mode by means of dynamic mechanical thermal spectrometer (Eplexor 150 N, Gabo Qualimeter, Ahlden, Germany). The temperature sweep test was performed at a frequency of 10 Hz and in a temperature range from −100 °C to 150 °C at a constant heating rate of 2 °C/min. The specimen was subject to a static pre-strain of 1% and oscillated with a dynamic strain of 0.5% for the dynamic mechanical measurements.

#### 2.2.6. Preparation of Rubber/PUU Blends

**Figure 1:**
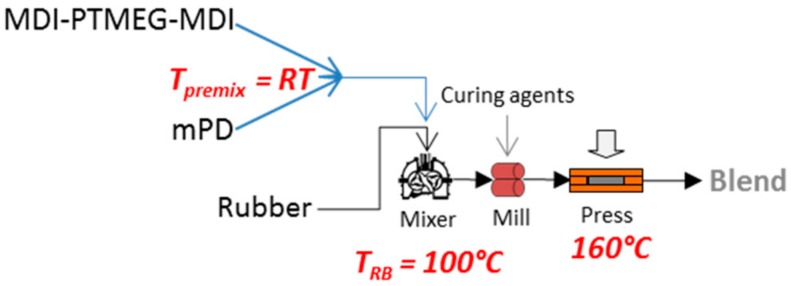
Reactive blending process to prepare rubber/PUU blends. The structures of PTMEG-MDI based isocyanate-terminated prepolymer and m-phenylene diamine are shown [23,24].

Figure 1 shows the reactive blending process opted to blend various rubbers with in situ synthesized polyurethane-urea in a fixed 70/30 weight ratio. The processing parameters, i.e., temperature of premixing (*T_premix_*) and temperature of reactive blending (*T_RB_*), were obtained from the chemo-rheological analysis of a prepolymer–diamine premix, as described in our previous work [23,24]. Importantly, the selected prepolymer and diamine develop a reactive premix, which do not react at the temperature of premixing (*T_premix_*) but at the temperature of reactive blending (*T_RB_*) in an internal mixer. In the beginning, polar or non-polar rubber was masticated in internal mixer and then a premix, composed of a liquid prepolymer and powder diamine, was poured into the mixing chamber. The reactive blending of rubber with in situ produced PUU was performed at *T_RB_* = 100 °C and speed of 70rpm. During blending, the reactive isocyanate groups of MT2184 and isocyanate reactive amino groups of mPD reacted to generate PUU in a rubber matrix. The progress of addition polymerization to PUU and homogenization of blend components was witnessed through the time-torque profile of blending procedure in an internal mixer. The prepared blends, according to the kind of rubber matrix, are compounded with curatives (peroxide, sulfur, metallic oxide, etc.) on a mixing mill at 50 °C. EPDM was cured with peroxide, CR with vulcanizing oxides (ZnO and MgO) and NR, XNBR and sSBR with sulfur-based curatives. Table 1 shows the formulations of compounds wherein the curatives are weighed as parts per hundred parts of rubber (phr). The blends were vulcanized by compression molding in a press at 160 °C and to their optimum cure time.

## 3. Results and Discussion

### 3.1. Curing Study 

**Figure 2:**
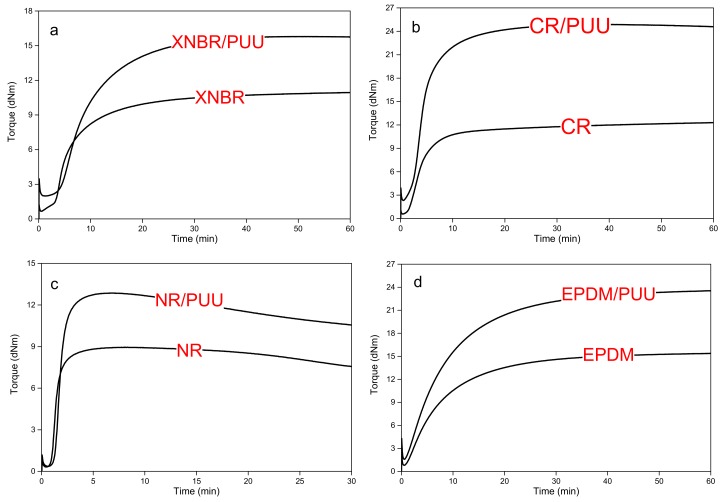

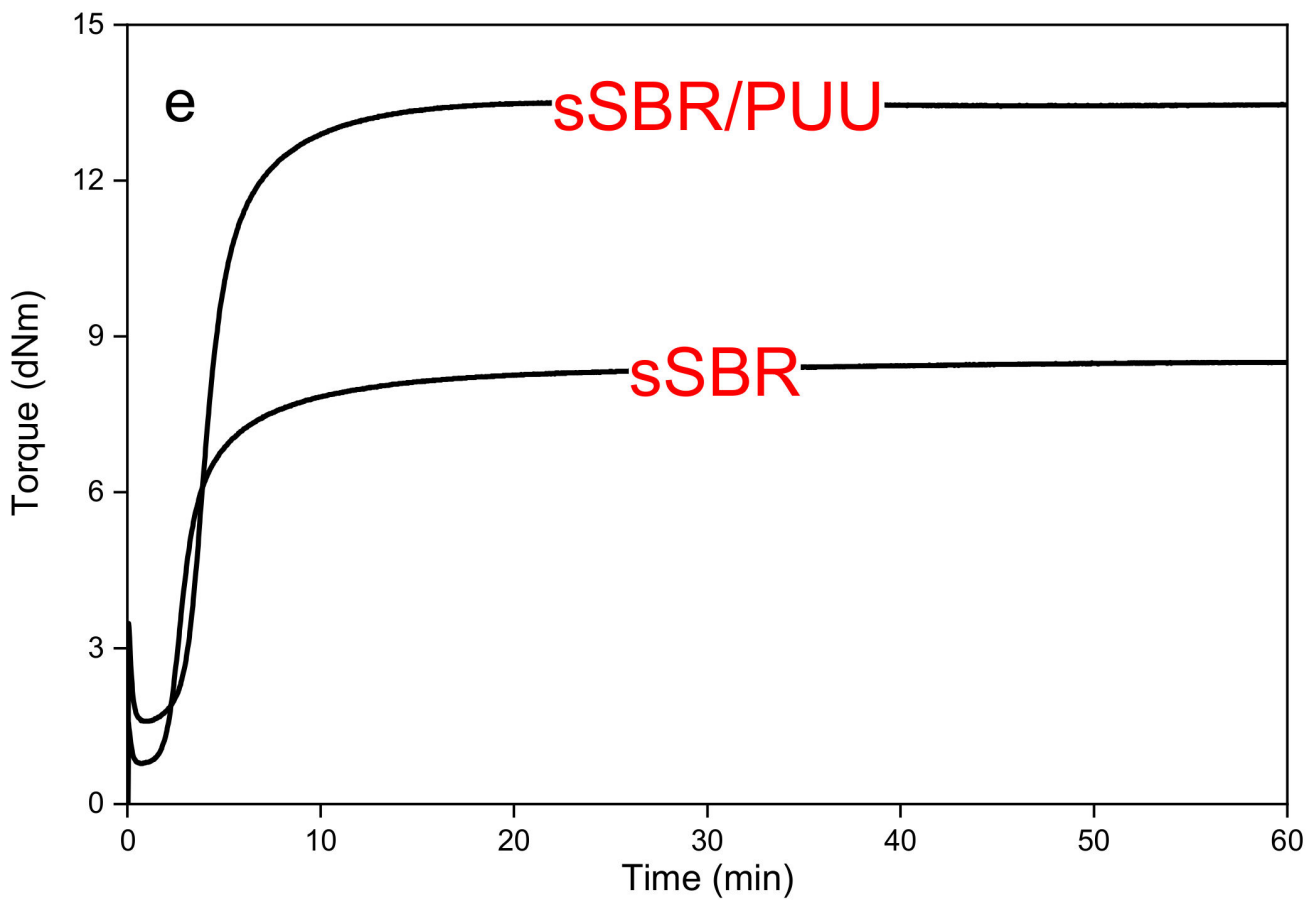
Vulcameter curves of blends are compared with the corresponding neat rubbers: (**a**) XNBR and XNBR/PUU; (**b**) CR and CR/PUU; (**c**) NR and NR/PUU; (**d**) EPDM and EPDM/PUU and (**e**) sSBR and sSBR/PUU.

The cure curves of compounds are compared in Figure 2. The values of minimum (*M_L_*), maximum (*M_H_*) and delta torques (*M_H_* − *M_L_*) are significantly higher for all the blends than rubbers. Interestingly, the value of *M_L_* is quite similar for both NR and NR/PUU. Neat NR was compounded with sulfur based curing system on a mixing mill at 50 °C and the NR/PUU blend was prepared in an internal mixer at around 100 °C prior to the addition of sulfur curatives, as shown in Figure 1. The thermo-mechanical history is an essential performance parameter for the NR [25,26,27]. The long molecular chains of NR are subjected to mechanical and thermo-oxidative breakdown in an internal mixer for the preparation of NR/PUU blend. NR with short molecular chains appears to lower the *M_L_* value of its blend with in situ PUU. PUU reinforces NR, however the scission of high molecular rubber chains to shorter ones subsides this reinforcement. 

All compounds develop a plateau cure curve except for the NR system (see Figure 2c). The cure curves of NR based compounds reach a maximum value and, thereon, exhibit reversion. This is typical for sulfur-cured NR compounds due to the non-oxidative aging on shearing at high temperatures which breaks crosslinks on over-curing [28,29].

The stiff PUU phase appears to restrict the mobility of rubber chains during vulcametric testing and brings about a greater delta torque value for all blends. The delta torque value relates to the crosslink density and blends show higher crosslink density than neat rubbers. PUU phase withstands the isothermal vulcanization temperature of 160 °C and does not soften. It acts as a solid inclusion similar to a conventional reinforcing silica or carbon black. 

### 3.2. Performance of Blends Based on Polar Rubbers

#### 3.2.1. Stress-Strain Response

**Figure 3:**
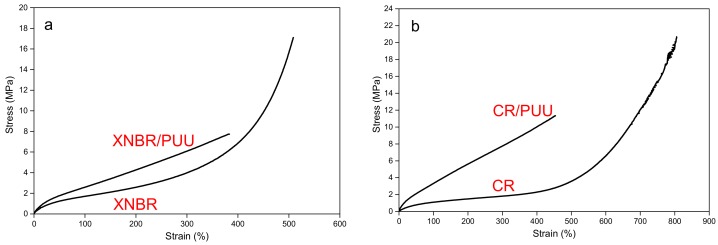
Stress–strain plots of vulcanizates: (**a**) XNBR and XNBR/PUU; and (**b**) CR and CR/PUU.

The in situ PUU improves the stress-strain response of neat XNBR and CR in a low-strain region, as is clear in Figure 3a,b, respectively. Both XNBR/PUU and CR/PUU take higher stresses than the corresponding neat rubbers for a similar deformation level (see Table 2), which is ascribed to an effective stress transfer between phases of heterogeneous blends. 

The stress upturn at large deformations impart superior ultimate tensile properties to XNBR and CR as compared to XNBR/PUU and CR/PUU respectively. The stress-strain response of XNBR relies on the formation of ionic clusters, wherein the pendant carboxyl groups are ionically linked by zinc oxide molecules [30,31,32,33,34,35]. In situ PUU significantly reinforces the nitrile rubber at low strain region (0–400%) but the ultimate tensile properties of XNBR/PUU are compromised. The decrease in the ultimate properties of XNBR/PUU blend is attributed either to the unavailability of carboxyl groups due to their possible reaction with isocyanate groups of pre-polymer during blending [36] or to the hindered mobility of rubber chains is presence of PUU phase which restrains the formation of ionic clusters or to the limited stretchability of nitrile rubber chains in presence of stiff PUU inclusions. 

CR shows strain induced crystallization at large strains, which contributes in improving the ultimate tensile properties [37,38,39,40]. In situ PUU reinforces the stress-strain curve of CR up to 450% strain but the ultimate tensile characteristics of CR/PUU blend are impaired. This is referred either to the hindrance in strain induced crystallization or to the limited stretchability of CR chains in presence of PUU phase.

It can be observed from the stress-strain plots of Figure 3a,d that the ultimate tensile failure seems to occur across the cross–section of test specimen for XNBR/PUU and CR/PUU blends (also discussed in Section 3.3.1). 

#### 3.2.2. Dynamic Temperature Sweep Study

**Figure 4:**
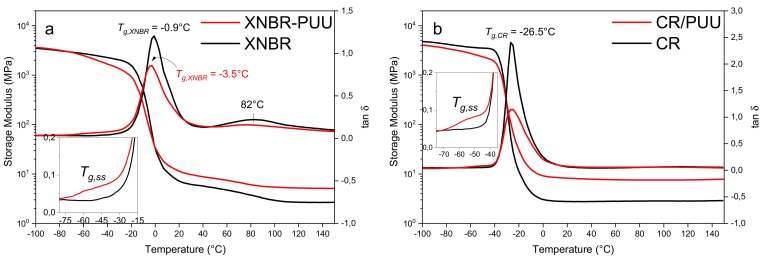
Storage modulus and tan δ as a function of temperature: (**a**) XNBR and XNBR/PUU; and (**b**) CR and CR/PUU.

The plots of storage modulus (E′) and loss factor (tan δ) as a function of temperature are shown in Figure 4 for polar rubbers and their blends with in situ PUU. Both XNBR/PUU and CR/PUU display two glass transition peaks in tan δ plot. The relaxation peak at approximately −50 °C corresponds to the glass transition temperature (*T_g,ss_*) of SS of PUU phase. The relaxation peaks at −0.9 °C and −26.5 °C correspond to the glass transition temperatures of XNBR and CR, respectively. XNBR exhibits a third relaxation peak at around 80 °C corresponding to the thermal dissociation of multiplets formed from the ionic association of carboxylic groups with divalent ZnO [35,41]. The formation and dissociation of multiplets is well established in Basu et al. [35]. These multiplets (ionic aggregates) act as thermally liable crosslinks and cause a tan δ hump in the rubbery plateau region of XNBR. In XNBR/PUU blend, the glass transition of XNBR is shifted downwards to −3.5 °C. The lowering of glass transition temperature is referred to the decrease in the density of ionic aggregates, as discussed in Section 3.2.1. Low concentration of ionic aggregates is beneficial in reducing the hysteresis loss and heat built up in XNBR/PUU blend when compared with XNBR. The low intensity of third tan δ hump in the rubbery plateau region of XNBR/PUU also supports this finding. 

The reinforcing PUU lowers the height of main tan δ peak of the rubber matrix. Table 3 shows that the tan δ peak height reduces from 1.2 to 0.9 for XNBR and from 2.4 to 1.1 for CR in blends. Contrary to earlier studies [8,9,10,11,12,13,14,15,16], the blends of present study exhibit a stable rubbery plateau region up to 150 °C due to the thermal stability of strong bidentate hydrogen bonded PUU phase [2]. 

The static and dynamic-mechanical performance of blends is superior to neat rubbers, which relates to the compatibility between blend phases. 

### 3.3. Performance of Blends Based on Nonpolar Rubbers

#### 3.3.1. Stress-Strain Response

**Figure 5:**
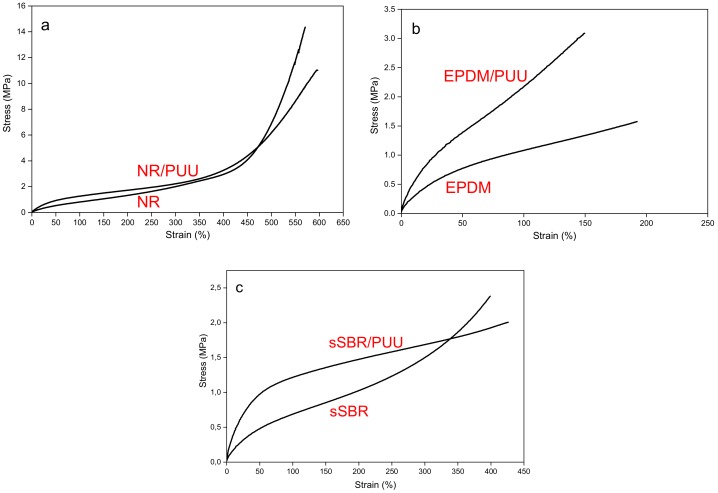
Stress-strain plots of non-polar rubbers and their blends: (**a**) NR and NR/PUU; (**b**) EPDM and EPDM/PUU; and (**c**) sSBR and sSBR/PUU.

The stress-strain curves of non-polar rubbers and their blends with in situ PUU are shown in Figure 5. An upward shift of the uniaxial tensile profile at low strain range is observed for all blends. In NR, the strain induced crystallization causes a steep rise in stress value at around 450% strain [42,43,44]. In NR/PUU blend, the strain facilitated crystallization is hindered by the presence of PUU phase and a sluggish rise of the stress values is recorded. The shortening of natural rubber chains during blending is also associated to the slack tensile response of NR/PUU blend. The strain hardening is responsible for the high tensile strength of sSBR as compared to sSBR/PUU blend [45]. Compared to neat sSBR, the stress-strain response of sSBR/PUU blend is significantly reinforced in a low strain region (see Figure 5c). However, beyond about a 50% strain of blend specimen, the rise in stress appears to be sluggish until a stress crossover at 340% strain and an ultimate stress at failure is reached. This unexpected stress-strain response of the blend suggests failure at the interface prior to the rubber matrix and, as a consequence, a lower value of ultimate strength is observed for the PUU reinforced sSBR than the neat sSBR. Such a uniaxial tensile response is not observed for any other rubber (excluding the strain-induced crystallizable NR). The in situ PUU imparts a significant improvement to the stress-strain response of neat EPDM, which is attributed, in addition to the strong interfacial adhesion, to the interfacial peroxide co-vulcanization of EPDM and PUU phases. 

The tensile test curves of the non-polar rubber based blends indicate that the failure occurs across the cross-section of test specimens of EPDM/PUU, NR/PUU, XNBR/PUU and CR/PUU. On the contrary, the creation of interfacial delaminating cracks during stretching is the reason of unusual tensile failure of sSBR/PUU blend.

#### 3.3.2. Dynamic Temperature Sweep Study

**Figure 6:**
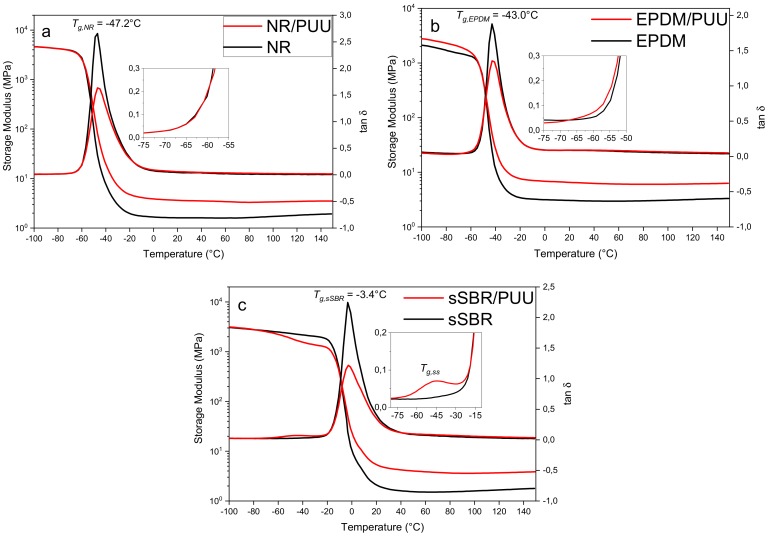
Tan δ and storage modulus versus temperature curves: (**a**) NR and NR/PUU; (**b**) EPDM and EPDM/PUU; and (**c**) sSBR and sSBR/PUU.

Figure 6a,b shows that both NR/PUU and EPDM/PUU exhibit only a single glass transition peak corresponding to the rubber phase. This is because of the overlapping of chain relaxation regions of rubber and SS of PUU. The tan δ curve of sSBR/PUU blend shows two glass transition peaks (see Figure 6c). The transition peak at approximately −50 °C (*T_g,ss_*) corresponds to the SS of PUU phase and at −3.4 °C (*T_g,sSBR_*) corresponds to the sSBR matrix of blend. 

Blends exhibit a high and stable rubbery plateau modulus as compared to the neat non-polar rubbers (see Table 3). In addition, the overlapping tan δ plots in the rubbery plateau region indicate a nearly equal dissipation of applied energy by rubbers and their blends with in situ PUU. 

The decrease in tan δ peak height of main transition and increase in the rubbery plateau modulus shows the potential of in situ PUU to reinforce both the polar and non-polar rubbers. Contrary to earlier studies [8,9,10,11,12,13,14,15,16], the PUU phase of new blends retains its rigidity and reinforcing capability up to a high temperature of 150 °C.

**Figure 7:**
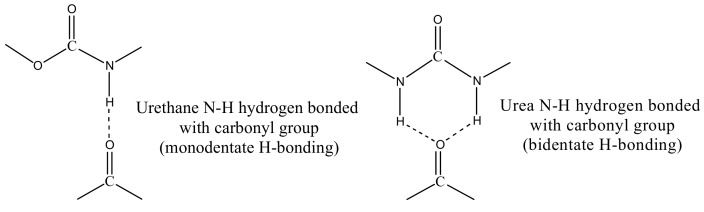
Mono- and bidentate hydrogen bonding between hard segments of in situ synthesized PUU of blends.

PUU phase retains stiffness even at high temperatures, which relates to the strong H-bonding interactions amongst hard segments. The urethane moieties form mono-dentate, whereas the urea moieties form bidentate hydrogen bonds. The bidentate hydrogen bonding is very strong and dissociate close to the temperature of polymer decomposition [46,47,48,49]. Urea hard segments remain intact through strong bidentate H-bonding and, consequently, blends can withstand a very high application temperature. The dynamic-mechanical testing of all the blends reflects that the stiffness and reinforcing tendency of PUU domains remains unaltered at high temperatures up to 150 °C. Yilgör, E. et al. [50] investigated model urea-urea and urethane-urethane systems by quantum mechanical calculations to provide a quantitative explanation to the H-bonding interactions. He found that the strongest hydrogen bonding interactions of 21.8 kJ/mol exist in urea-urea systems with bidentate H-Bonding as compared to the value of 18.4 kJ/mol in urethane-urethane systems with monodentate H-Bonding (see Figure 7). 

### 3.4. Evidence of Compatibility in Rubber/PUU Blends

#### 3.4.1. SEM-EDX Analysis

**Figure 8:**
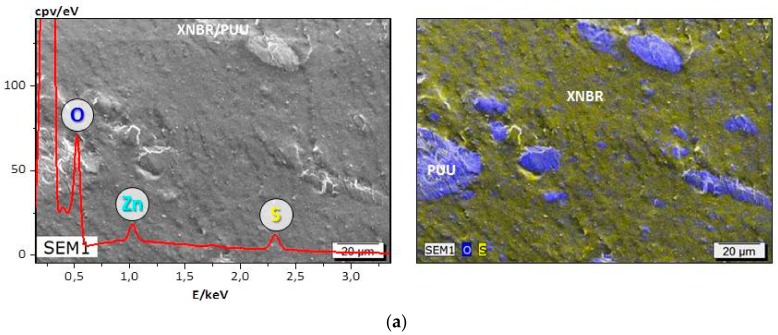

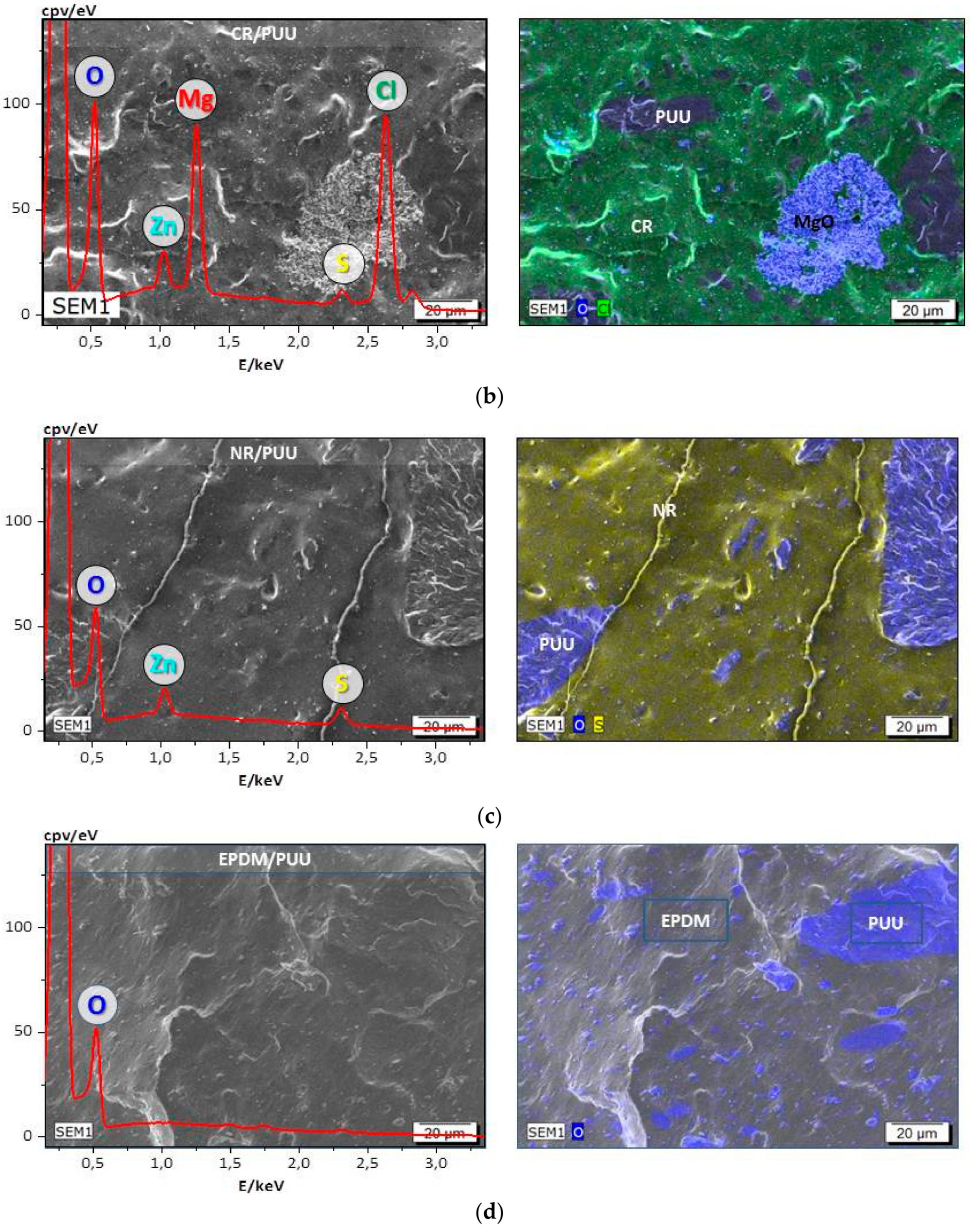

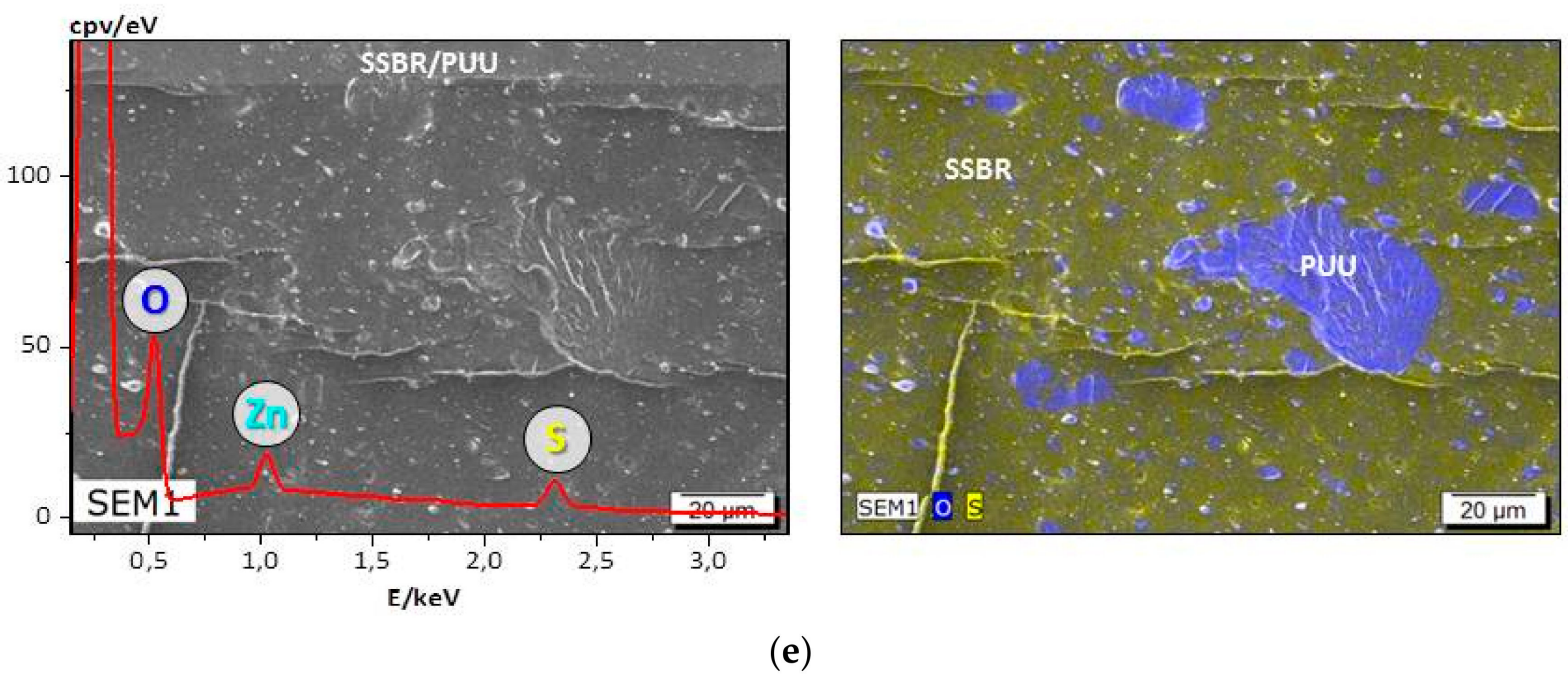
SEM, SEM-EDX images along with the respective EDX spectrum of: (**a**) XNBR/PUU; (**b**) CR/PUU; (**c**) NR/PUU; (**d**) EPDM/PUU; and (**e**) sSBR/PUU blends. Elemental oxygen mapping (O) identifies PUU domains in blends.

The EDX spectra, and SEM and SEM-EDX images of cryogenically fractured surfaces of blend specimens are shown in Figure 8. No voids are observed around the dispersed PUU domains, which are strongly adhered to the surrounding rubber matrix. The phases of heterogeneous blends are not distinguishable by SEM alone; however, an elemental oxygen mapping with energy dispersive X-ray spectroscopy identifies PUU domains as oxygen-rich areas in blue color. The irregularity of domains is ascribed to the reality that the very fast polyaddition reactions of isocyanates with amino groups produce hard segments during the reactive blending procedure in internal mixer. The in situ generated hard segments associate spontaneous with each other by strong bidentate hydrogen bonding to freeze the actual irregular appearance of PUU domains. Importantly, due to the aromatic nature and high content of H-bonded hard segments, the irregular PUU domains are not flexible and cannot be reshaped to be of spherical geometry of minimal surface area. The EDX analysis identifies surface elemental oxygen, sulfur, zinc, magnesium and chlorine. Sulfur and zinc oxide are part of curing system used to crosslink XNBR, NR and sSBR, therefore, the corresponding EDX spectra of Figure 8a,c,e shows peaks of elemental S and Zn. MgO and ZnO are used to cure CR and the characteristic spectral peaks of elemental Zn, Mg, S and Cl are recorded in the EDX spectrum in Figure 8b. In addition, the regions of agglomerated MgO are identified in SEM-EDX image of CR/PUU blend. It is observed that the curing chemicals are present only in the rubber phase of blends. This reflects that the migration of curing chemicals into the PUU phase is inhibited by the stiffness of domains. The SEM-EDX analysis shows that the interface between PUU and rubber remains strongly adhered in all blends. Regardless of rubber polarity, the entrenched interfacial adhesion between two distinct phases reflects existence of a mutual interpenetrated and mechanical interlocked interfacial region of polymer chains.

#### 3.4.2. Energy-Filtered Transmission Electron Microscopy

**Figure 9:**
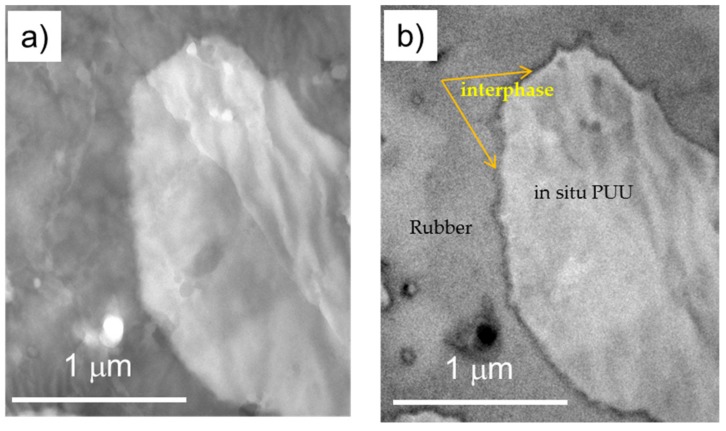
TEM images of nitrile rubber based blend: (**a**) bright field image; and (**b**) carbon map.

**Figure 10:**
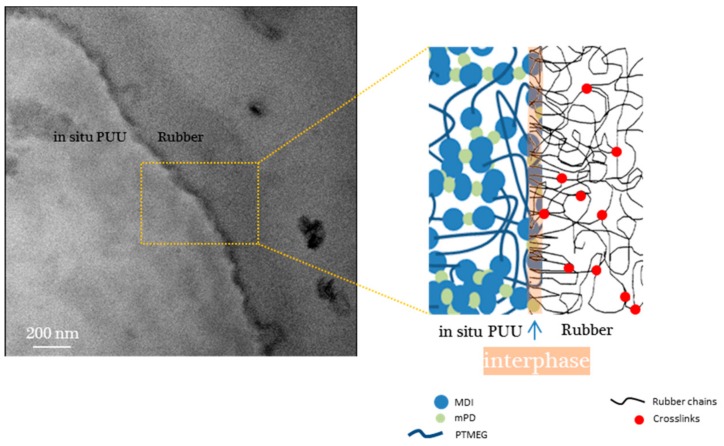
TEM image of nitrile rubber based blend with a graphical representation of interphase region between the cured rubber and in situ synthesized PUU.

The dispersed phase morphology of nitrile rubber based heterogeneous blend is illustrated in Figure 8c and Figure 9. The circular-cum-elliptical and different micro-sized PUU domains are found embedded in the rubber matrix, as reported in [24]. A couple of dark regional spots are also visible in rubber matrix, which are probably ZnO particles. In a bright field image (Figure 9a), the brighter discrete area corresponds to PUU and the darker continuous area corresponds to the nitrile rubber. TEM image in Figure 9b shows the elemental carbon distribution where a thin interphase layer is visible as a dark borderline along the interface between distinct phases. The bright and dark field TEM images in Figure 9 are exemplary for other blends. A very dark interface region suggests that the transmission of incident electrons is hindered due to a relatively compact composition of atoms at the interphase. The exact chemical composition of interphase is not easy to determine; however, its grayscale intensity suggests a compact region of high electron density due to the interdiffusion and intermingling of distinct polymer chains. The interphase region is formed from the mechanical interlocking of mutually interpenetrated chains of both rubber and PUU polymers. The reactive blending procedure, wherein the synthesis of polyurethane-urea via a viscous prepolymer precursor is accomplished in an internal mixer, realizes the incursions of polyurethane-urea chains into the rubber fissures to create a region of mutually entangled chains termed as an interphase in Figure 10. 

## 4. Conclusions

The reactive blending of a stiff polyurethane-urea with different rubbers was successfully realized in an internal mixer at a low temperature of 100 °C. The formation of interfacial entanglements between polymer chains during the reactive blending procedure manifests itself by enhancing the tensile and dynamic-mechanical properties of all rubber/PUU blends. Blends retain their reinforced property spectrum up to a high temperature of 150 °C, as evident from the temperature sweep tests. SEM-EDX reveals a stronger interfacial adhesion between distinct phases of all the heterogeneous rubber/PUU blends. EFTEM analysis proves the formation of an interphase region between the blend phases. It could be shown that the in situ generation of PUU in elastomer polymers can be successfully carried out in both polar and non-polar rubbers. This leads to wide application perspectives for technical elastomers, e.g., belts, rollers or seals, as well as tires.

## 5. Patents

The described in situ blending process and the obtained materials is patent pending: Blends aus thermoplastischen Polyurethanen und Kautschuk und Verfahren zu ihrer Herstellung Tahir, M., Mahmood, N., Stöckelhuber, K.W., Heinrich G., Das, A., Jurk R. (Inventors)Applicant: Leibniz-Institut für Polymerforschung Dresden e.V.German Patent application: DE 102013217661;also published as: WO 2015/032681 A1, US2016194483 (A1), KR20160056906, JP2016529376A(6184 491), EP3041877 (A1).

## Figures and Tables

**Table 1:** Formulation of compounds in weight parts per hundred parts of rubber (phr).

Ingredients	Rubbers					Rubber/PUU Blends				
	NR	sSBR	XNBR	CR	EPDM	NR/PUU	sSBR/PUU	XNBR/PUU	CR/PUU	EPDM/PUU
Rubber	100	100	100	100	100	70	70	70	70	70
Premix	-	-	-	-	-	30	30	30	30	30
ZnO	3	3	3	5	-	2.1	2.1	2.1	3.5	-
Stearic Acid	2	2	2	-	-	1.4	1.4	1.4	-	-
DPG	2	2	2	-	-	1.4	1.4	1.4	-	-
CBS	1.5	1.5	1.5	-	-	1.1	1.1	1.1	-	-
Sulfur	1.5	1.5	1.5	-	-	1.1	1.1	1.1	-	-
MgO	-	-	-	4	-	-	-	-	2.8	-
Peroxide	-	-	-	-	3	-	-	-	-	2.1
Coagent	-	-	-	-	2	-	-	-	-	1.4

**Table 2:** Tensile characteristics of neat Rubbers and their Rubber/PUU blends.

Compound	Young’s Modulus (MPa)	Modulus at 100% Elongation (MPa)	Tensile Strength (MPa)	Elongation at Break (%)
NR	1.33 ± 0.17	0.83 ± 0.01	14.43 ± 1.20	570 ± 8
NR/PUU	3.30 ± 0.15	1.26 ± 0.01	11.04 ± 0.09	595 ± 4
XNBR	4.79 ± 0.12	1.71 ± 0.01	20.64 ± 5.01	525 ± 24
XNBR/PUU	6.81 ± 0.53	2.63 ± 0.05	7.08 ± 0.08	373 ± 14
sSBR	1.63 ± 0.01	0.69 ± 0.01	2.48 ± 0.28	403 ± 16
sSBR/PUU	3.89 ± 0.44	1.20 ± 0.01	2.00 ± 0.03	425 ± 10
CR	2.72 ± 0.10	1.10 ± 0.02	20.68 ± 2.10	805 ± 18
CR/PUU	8.75 ± 0.25	3.31 ± 0.03	11.72 ± 0.54	470 ± 23
EPDM	2.43 ± 0.17	1.08 ± 0.01	1.65 ± 0.10	205 ± 18
EPDM/PUU	5.12 ± 0.10	2.16 ± 0.02	3.16 ± 0.10	154 ± 7

**Table 3:** Dynamic-mechanical characteristics of neat rubbers and their rubber/PUU blends.

Compound	Glass Transition Temperature of Rubber *T_g,rubber_* (°C)	tan δ Peak Height at *T_g,rubber_*	Storage Modulus at 25 °C (MPa)
NR	−47	2.7	1.7
NR/PUU	−46	1.6	3.6
XNBR	−1	1.2	6.5
XNBR/PUU	−4	0.9	10.2
sSBR	−3	2.3	1.9
sSBR/PUU	−3	1.2	4.8
CR	−27	2.4	2.8
CR/PUU	−27	1.1	8.0
EPDM	−43	1.9	3.1
EPDM/PUU	−43	1.4	6.4
